# The effect of framing and communicating COVID-19 vaccine side-effect risks on vaccine intentions for adults in the UK and the USA: A structured summary of a study protocol for a randomized controlled trial

**DOI:** 10.1186/s13063-021-05484-2

**Published:** 2021-09-06

**Authors:** Nikkil Sudharsanan, Caterina Favaretti, Violetta Hachaturyan, Till Bärnighausen, Alain Vandormael

**Affiliations:** 1 Heidelberg Institute of Global Health, Heidelberg University, Heidelberg, Germany; 2 Department of Global Health and Population, Harvard T. H Chan School of Public Health, Boston, MA USA; 3 Africa Health Research Institute (AHRI), Somkhele, KwaZulu-Natal South Africa

**Keywords:** COVID-19, risk communication, vaccine hesitancy, behavioral science, randomized controlled trial, factorial designs, public healthinternet-based studies, protocol

## Abstract

**Objectives:**

Vaccine hesitancy is a major hurdle for stopping the COVID-19 pandemic. Recently, fear of vaccine side effects created widespread concern and paused global vaccination efforts. Many studies find that how medical risks are framed and communicated can influence individuals' perceptions and behavior, yet there is little evidence on how the communication of COVID-19 vaccine side-effect risks influences vaccine intentions. The primary objective of our study is to evaluate how the framing of vaccine-side effect risks impacts individuals' vaccine intentions and perceptions of vaccine safety. The study will assess the impact of 3 dimensions of side-effect framing:

1. *Qualitative risk labels:* Determine whether attaching a qualitative risk label (e.g. adding "very low risk" next to the actual numerical risk) impacts individuals' willingness to take a vaccine and their perceptions of its safety.
2. *Comparison groups:* Determine how framing side-effect risks in comparison to other causes of mortality (COVID-19 mortality and motor vehicle mortality) impacts individuals’ willingness to take a vaccine and their perceptions of its safety.
3. *How the comparison risks are presented:* Determine whether comparisons to other causes of mortality are presented on an absolute or relative scale impacts individuals’ willingness to take a vaccine and their perceptions of its safety.

Secondarily, we will also randomize a subset of individuals to receive the "status-quo" framing, where the vaccine side-effect risks are presented like how they were presented in the media. We will then compare vaccine intentions and perceptions of vaccine safety between the status-quo and the pooled intervention group samples to provide some insight into how "harmful" the status-quo framing was. Ultimately, we believe that our results will provide the some of the first experimental evidence on how the communication of COVID-19 vaccine risks may impact the public's willingness to be vaccinated and can inform future efforts to increase vaccination rates.

**Trial design:**

Our study is an online-based randomized controlled trial designed to evaluate the effect of different vaccine side-effect framings on COVID-19 vaccine intentions and perceived safety for a hypothetical COVID-19 vaccine. Using a factorial design, we will experimentally assess the impact of 3 risk framing strategies, varying whether the risk is presented: (1) with a qualitative label, (2) whether the risk is presented with a comparison risk, and (3) for comparison cases, whether the comparison is in absolute or relative terms. We will also randomize a portion of respondents to a status quo framing where the side effect risk mimics the media's communication in early April 2021.

**Participants:**

This will be an online study setting. We will use Prolific to recruit participants and host our study on the Gorilla platform. To be eligible, participants must be 18 years old or over (male, female, or other), have current residence in the US or UK, and be able to speak English. Participants will be excluded from the study if they do not meet our inclusion criteria.

**Intervention and comparator:**

Our study content will consist of five pages presented to individuals online. Page 1 will explain the purpose of the study and contain the consent information. Page 2 will contain basic sociodemographic questions, including participants' age, sex, and schooling level. Page 3 will set up the experiment by telling individuals that we will describe a hypothetical new COVID-19 vaccine and that we would like to know how likely they would be to take the vaccine and how safe they think the vaccine is. On this page, we will also encourage individuals to respond truthfully and remind them that their answers are confidential and cannot be linked back to any personal identifying information. Page 4 will be the main experimental slide, where we will present individuals with information on the vaccine, varying how the vaccination risk is communicated based on which experimental framing arm they are randomized to.

We will factorially randomize across the following factors in the following order (separately by country). First, we will determine whether individuals are randomized to the status quo framing, or the intervention framings (1500 respondents to the status quo, and 4500 to the intervention). Among those randomized to the intervention framing, we will randomize (equal allocation) whether the side effect is presented without a comparison, with a comparison to COVID-19 mortality, or with a comparison to motor vehicle mortality. We will then factorially randomize (equal allocation) whether the risk is presented with a qualitative risk label or not (e.g. "very low risk"). To ensure that the factors are independent of one another, we will do this by randomizing individuals to the risk labels within strata of the comparison group factor. Lastly, among those randomized to the comparison group, we will factorially randomize whether the risk is presented as an absolute or relative comparison. As previously, we will ensure independence by doing this randomization within strata of comparison group*risk labelling. This entire design is visualized in the full protocol.

The experimental text for each arm is:

Arm 1: With regards to side effects, so far 8 individuals have developed potentially life-threatening blood clots. This is among the approximately 7 million adults that have received the vaccine so far.

Arm 2: With regards to side effects, 1 out of 100,000 vaccinated individuals may develop serious blood clots.

Arm 3: With regards to side effects, 1 out of 100,000 vaccinated individuals may develop serious blood clots (very low risk).

Arm 4:

- *Text for USA participants:* With regards to side effects, 1 out of 100,000 vaccinated individuals may develop serious blood clots. As a reference, 170 out of every 100,000 unvaccinated Americans died of COVID-19 based on data from the past year.
- *Text for UK participants:* With regards to side effects, 1 out of 100,000 vaccinated individuals may develop serious blood clots. As a reference, 108 out of every 100,000 unvaccinated individuals in the UK died of COVID-19 based on data from the past year.

Arm 5:

- *Text for USA participants:* With regards to side effects, 1 out of 100,000 vaccinated individuals may develop serious blood clots. As a reference, this is 1/170th of the risk of COVID-19 mortality among unvaccinated Americans based on data from the past year.
- *Text for UK participants:* With regards to side effects, 1 out of 100,000 vaccinated individuals may develop serious blood clots. As a reference, this is 1/108th of the risk of COVID-19 mortality among unvaccinated individuals in the UK based on data from the past year.

Arm 6:

- *Text for USA participants:* With regards to side effects, 1 out of 100,000 vaccinated individuals may develop serious blood clots (very low risk). As a reference, 170 out of every 100,000 unvaccinated Americans died of COVID-19 based on data from the past year.
- *Text for UK participants:* With regards to side effects, 1 out of 100,000 vaccinated individuals may develop serious blood clots (very low risk). As a reference, 108 out of every 100,000 unvaccinated individuals in the UK died of COVID-19 based on data from the past year.

Arm 7:

- *Text for USA participants:* With regards to side effects, 1 out of 100,000 vaccinated individuals may develop serious blood clots (very low risk). As a reference, this is 1/170th of the risk of COVID-19 mortality among unvaccinated Americans based on data from the past year.
- *Text for UK participants:* With regards to side effects, 1 out of 100,000 vaccinated individuals may develop serious blood clots (very low risk). As a reference, this is 1/108th of the risk of COVID-19 mortality among unvaccinated individuals in the UK based on data from the past year.

Arm 8:

- *Text for USA participants:* With regards to side effects, 1 out of 100,000 vaccinated individuals may develop serious blood clots. As a reference, 12 out of every 100,000 Americans died in a motor vehicle accident based on data from the past year.
- *Text for UK participants:* With regards to side effects, 1 out of 100,000 vaccinated individuals may develop serious blood clots. As a reference, 2.6 out of every 100,000 individuals in the UK died in a motor vehicle accident based on data from the past year.

Arm 9:

- *Text for USA participants:* With regards to side effects, 1 out of 100,000 vaccinated individuals may develop serious blood clots. As a reference, this is 1/12th of the risk of dying in a motor vehicle accident based on data from the past year.
- *Text for UK participants:* With regards to side effects, 1 out of 100,000 vaccinated individuals may develop serious blood clots. As a reference, this is almost 1/4th of the risk of dying in a motor vehicle accident based on data from the past year.

Arm 10:

- *Text for USA participants:* With regards to side effects, 1 out of 100,000 vaccinated individuals may develop serious blood clots (very low risk). As a reference, 12 out of every 100,000 Americans died in a motor vehicle accident based on data from the past year.
- *Text for UK participants:* With regards to side effects, 1 out of 100,000 vaccinated individuals may develop serious blood clots (very low risk). As a reference, 2.6 out of every 100,000 individuals in the UK died in a motor vehicle accident based on data from the past year.

Arm 11:

- *Text for USA participants:* With regards to side effects, 1 out of 100,000 vaccinated individuals may develop serious blood clots (very low risk). As a reference, this is 1/12th of the risk of dying in a motor vehicle accident based on data from the past year.
- *Text for UK participants:* With regards to side effects, 1 out of 100,000 vaccinated individuals may develop serious blood clots (very low risk). As a reference, this is nearly 1/4th of the risk of dying in a motor vehicle accident based on data from the past year.

The risk information will be presented on a single page along with the two main outcome questions. Lastly, for individuals that reported that they are unlikely or unsure about whether they would take the vaccine, the final page will ask them their reason (question based on a recently published study of COVID-19 vaccine hesitancy).

**Main outcomes:**

Our primary outcome is individuals' willingness to take the hypothetical COVID-19 vaccine. We will measure this outcome by asking, "How likely would you be to take this vaccine?" allowing individuals to choose from a four-point Likert response of "Unlikely, Unsure leaning towards unlikely, Unsure leaning towards likely, Very likely." This outcome variable, including the categories and phrasing, is based on a recently published study on COVID-19 vaccine hesitancy conducted by researchers with the Vaccine Hesitance Project at the London School of Hygiene and Tropical Medicine. Our secondary outcome is individuals' perceived safety of the vaccine. We will assess this outcome by asking individuals, "How safe do you feel this vaccine is?" allowing them to choose answers ranging from 1-10 where 1 is extremely unsafe, and 10 is extremely safe. Both outcomes will be measured at the time of the questionnaire. Participants can take up to 45 min to complete the questions but will not be able to go back and change their responses after submitting their questionnaire.

**Randomization:**

Using a web-based randomization algorithm, Gorilla will randomly allocate participants to each of the experimental arms. Gorilla allows for two randomization options - independent randomization of each individual based on a probability draw and balanced randomization, which randomizes without replacement such that among groups of respondents a fixed proportion will end up in each experimental arm. We will use the "balanced randomization" option to ensure that our experimental arms are balanced. Participants will be randomized based on the allocations described above.

**Blinding:**

Because Prolific handles the interaction between the study investigators and participants, the participants will be completely anonymous to the study investigators. The outcome measures will be self-reported and submitted anonymously. All persons in the study team will be blinded to the group allocation.

**Numbers to be randomized:**

We will randomize 6000 participants per country for a total sample of 12000 individuals.

**Trial status:**

The protocol version number is 1.0 and the date is July 14, 2021. Recruitment is expected to begin on 26 July 2021 and end by August 10, 2021.

**Trial registration:**

The study and its outcomes were registered at the German Clinical Trials Register (www.drks.de) on July 12th, 2021: #DRKS00025551.

**Full protocol:**

The full protocol is attached as an additional file, accessible from the Trials website (Full_Protocol_20Jul2021) In the interest of expediting dissemination of this material, the familiar formatting has been eliminated; this Letter serves as a summary of the key elements of the full protocol.

**Supplementary Information:**

The online version contains supplementary material available at 10.1186/s13063-021-05484-2.

## Supplementary Information

**Additional file 1.** Full protocol.

## Data Availability

Data will be collected and stored on the Gorilla platform. The study investigators own and have complete control of the research data, which can be accessed at any time. For statistical analysis, the data will be downloaded and safely stored on a computing system maintained by the Heidelberg University.

