# Supplementary material for: The effect of framing and communicating COVID-19 vaccine side-effect risks on vaccine intentions for adults in the UK and the USA: A structured summary of a study protocol for a randomized controlled trial
Source: Trials. 2021 Sep 6;22:592. doi: 10.1186/s13063-021-05484-2 (PMC8419378; doi:10.1186/s13063-021-05484-2)
Supplement: Supplementary file 1 — Additional file 1. Full protocol. [file 13063_2021_5484_MOESM1_ESM.docx]

**Title**

The effect of framing and communicating COVID-19 vaccine side-effect risks on vaccine intentions: a randomized controlled tria**l**

**Names protocol contributors**

Nikkil Sudharsanan^1^, Caterina Favaretti^1^, Violetta Hachaturyan^1^, Till Bärnighausen^1,2,3^, Alain Vandormael^1^

**Abstract**

- **Background**: Vaccine hesitancy is a major hurdle for stopping the COVID-19 pandemic Recently, fear of vaccine side effects created widespread concern and paused global vaccination efforts. Many studies find that how medical risks are framed and communicated can influence individuals' perceptions and behavior, yet there is no evidence on how the communication of COVID-19 vaccine side-effect risks influences vaccine intentions.
- **Methods**: We propose conducting a randomized experiment to assess how the framing and communication of vaccine side-effect information affect individuals' vaccination intentions. We will recruit 12000 adults from the United States and United Kingdom through Prolific, a platform for conducting web-based studies. Individuals will be presented information about a hypothetical COVID-19 vaccine and asked how likely they would be to take the vaccine. Within this presentation, we will experimentally vary how the side-effect risk is communicated by randomizing whether the risk is presented with a qualitative label, what type of comparison the risk is presented with, and whether the comparisons are presented in an absolute or relative scale. We will then estimate the effect of different framings on vaccine intentions and individuals' perceptions of vaccine safety.
- **Discussion**: The results from this study will provide the first experimental evidence on how the communication of COVID-19 vaccine risks impacts willingness to be vaccinated and can inform ongoing vaccination efforts.

**Trial registration**: The study has been registered in the German Clinical Trials Register (www.drks.de) on 12 July 2021 (#DRKS00025551)

**Keywords**

COVID-19, vaccine hesitancy, risk communication, behavioral science

**Administrative information**

Note: the numbers in curly brackets in this protocol refer to SPIRIT checklist item numbers. The order of the items has been modified to group similar items (see <http://www.equator-network.org/reporting-guidelines/spirit-2013-statement-defining-standard-protocol-items-for-clinical-trials/>).

| Title {1} | The effect of framing and communicating COVID-19 vaccine side-effect risks on vaccine intentions: a randomized controlled trial |
| --- | --- |
| Trial registration {2a and 2b}. | Trial DRKS00025551 in the German Clinical Trials Register (www.drks.de) |
| Protocol version {3} | Version 1.0, 14 July 2021 |
| Funding {4} | This study is funded by the Heidelberg Institute of Global Health at the University of Heidelberg, Germany |
| Author details {5a} | ^1^ Heidelberg Institute of Global Health, University of Heidelberg, Heidelberg, Germany  ^2^ Department of Global Health and Population, Harvard T.H Chan School of Public Health, Boston, MA, USA  ^3^ Africa Health Research Institute (AHRI), Somkhele, KwaZulu-Natal, South Africa |
| Name and contact information for the trial sponsor {5b} | Prof. Dr. Dr. Till Bärnighausen, till.baernighausen@uni-heidelberg.de |
| Role of sponsor {5c} | Prof. Bärnighausen is a member of the study team and has provided key input into the study design and will also be involved in the interpretation of data and writing of the report. |

**Introduction**

**Background and rationale {6a}**

Vaccination is the main global strategy for halting the COVID-19 pandemic. In the United States (US) and United Kingdom (UK), despite the widespread availability of vaccines, overall rates of vaccination have stagnated.^1^ Just 59% of the US population and 52% of the UK population is fully vaccinated, with rates near 33% in the US South. Vaccination rates are even lower in many other countries, especially in low and middle-income countries like India where just 5.4% of individuals are fully vaccinated.^2^

Vaccine hesitancy is a major barrier to improving vaccination rates. There are many reasons that individuals may be unsure about vaccination, including concerns of safety or perceptions of effectiveness. Recently, fear of side effects related to the AstraZeneca and Johnson & Johnson vaccines created widespread concern and prompted pausing of vaccination efforts throughout many parts of the world. Although the side effects were extremely rare (an estimated 1 out of 100,000 people administered the AstraZeneca vaccine and 0.7 out of 100,000 administered the Johnson & Johnson vaccine developed serious blood clots),^3,4^ narratives around the side-effects likely amplified existing concerns and hesitancy.

There is a large literature in risk communication emphasizing that the way medicine benefits and risks are communicated to individuals can influence their perceptions and ultimately behavior.^5-7^ For example, an experiment of risk for a hypothetical cholesterol drug found that individuals' willingness varied depending on whether the risk was presented as a frequency or percentage and whether it carried a qualitative risk label or not.^8^ Similarly, a recent experimental study found that individuals that were presented information about the safety/efficacy of a vaccine were more likely to state that they would take it compared to individuals that were presented information that linked vaccine development to political forces.^9^

To the best of our knowledge, there was no communication strategy for informing the public about the side-effect risks of the AstraZeneca and Johnson & Johnson vaccines with risk information coming from multiple uncoordinated media sources. Developing and adhering to a vaccination communication plan will thus be essential for preventing confusion and hesitancy around future vaccination efforts. To date, however, there are no studies that have examined how the framing and communication of COVID-19 vaccine side-effect risks influence individuals' perceptions of vaccine safety and ultimately their vaccine intentions.

**Objectives {7}**

The general aim of the proposed study is to inform efforts to improve COVID-19 vaccination rates by generating rigorous scientific insights on how the communication of vaccine side-effect risks to the public influence their willingness to take a vaccine and their perceptions of vaccine safety. The primary objective of our study is to evaluate how the framing of vaccine-side effect risks impacts individuals' vaccine intentions and perceptions of safety. Using a factorially randomized experimental design with 12000 adults (6000 in the US and 6000 in the UK), our study will assess the impact of 3 dimensions of side-effect framing:

1. *Qualitative risk labels:* Determine whether attaching a qualitative risk label (e.g. adding "very low risk" next to the actual numerical risk) impacts individuals' willingness to take a vaccine and their perceptions of its safety

2. *Comparison groups:* Determine how framing side-effect risks in comparison to other causes of mortality (COVID-19 mortality and motor vehicle mortality) impacts individuals’ willingness to take a vaccine and their perceptions of its safety.

3. *How the comparison risks are presented:* Determine whether comparisons to other causes of mortality are presented on an absolute or relative scale impacts individuals’ willingness to take a vaccine and their perceptions of its safety.

Secondarily, we will also randomize a subset of individuals to receive the "status-quo" framing, where the vaccine side-effect risks are presented like how they were presented in the media. We will then compare vaccine intentions and perceptions of vaccine safety between the status-quo and the pooled intervention group samples to provide some insight into how "harmful" the status-quo framing was.

**Trial design {8}**

Our study is an online-based factorial trial.

**Methods: Participants, interventions and outcomes**

**Study setting {9}**

This will be an online study setting. We will use Prolific^10^ (a web-based service for recruiting participants for online studies) to recruit participants and host our study on the Gorilla platform (a platform for designing and conducting online behavioral science experience).^11^ To be eligible, participants must be 18 years old or over, have current residence in the US or UK, and be able to speak English. Participants will be excluded from the study if they do not meet our inclusion criteria..

**Eligibility criteria {10}**

Participants must be 18 years old or over, have current residence in the United States or United Kingdom, and be able to speak English.l

**Who will take informed consent? {26a}**

Participants will read and fill out an informed consent form through the Prolific platform themselves.

**Additional consent provisions for collection and use of participant data and biological specimens {26b}**

Not applicable.

**Interventions**

**Explanation for the choice of comparators {6b}**

Our main comparators are different ways of framing and communicating the side-effect risks of a hypothetical COVID-19 vaccine. We aim to factorially evaluate three framings, based on the existing literature on risk communication: (1) is the side-effect risk presented with a qualitative risk label? (2) is the side-effect risk presented with a comparison, and if so, are comparisons to COVID-19 mortality or motor vehicle mortality (a commonly used comparison in the broader literature) more salient? (3) among risks presented with a comparison, is the comparison presented in an absolute or relative way? Secondarily, we will also assign some individuals to a status-quo framing, where the side-effect risks are presented similarly to how they were presented by the media in early April. The aim of this last comparator is to determine to what extent *any* form informed risk communication might have affected vaccine intentions compared to how risks were actually presented to the public.

**Intervention description {11a}**
Our study content will consist of five pages of information presented to individuals online. Page 1 will explain the purpose of the study and contain the consent information. Page 2 will contain basic sociodemographic questions, including participants' age, sex, and schooling level. Page 3 will set up the experiment by telling individuals that we will describe a hypothetical new COVID-19 vaccine and that we would like to know how likely they would be to take the vaccine and how safe they think the vaccine is. On this page, we will also encourage individuals to respond truthfully and remind them that their answers are confidential and cannot be linked back to any personal identifying information. Page 4 will be the main experimental slide, where we will present individuals with information on the vaccine, varying how the vaccination risk is communicated based on which experimental framing arm they are randomized to.

We will factorially randomize across the following factors in the following order (separately by country). First, we will determine whether individuals are randomized to the status quo framing, or the intervention framings (1500 respondents to the status quo, and 4500 to the intervention). Among those randomized to the intervention framing, we will randomize (equal allocation) whether the side effect is presented without a comparison, with a comparison to COVID-19 mortality, or with a comparison to motor vehicle mortality. We will then factorially randomize (equal allocation) whether the risk is presented with a qualitative risk label or not (e.g. "very low risk"). To ensure that the factors are independent of one another, we will do this by randomizing individuals to the risk labels within strata of the comparison group factor. Lastly, among those randomized to the comparison group, we will factorially randomize whether the risk is presented as an absolute or relative comparison. As previously, we will ensure independence by doing this randomization within strata of comparison group*risk labelling. This entire design is visualized in Figure 1 (shown for just one country).

**Figure 1:** Trial design.

| **Randomization 1: Status quo vs. Intervention Arms** | |
| --- | --- |
| *Status-quo framing (N = 1500)* | *Intervention arms (N = 4500)* |
| *Arm 1* | *Follow the specific scripts chart below* |

| **Randomization 2: Which comparison group?** | | | | | | | | | |
| --- | --- | --- | --- | --- | --- | --- | --- | --- | --- |
| *No comparison (N = 1500)* | | *Covid-19 (N = 1500)* | | | | *Motor vehicle mortality (N = 1500)* | | | |
| **Randomization 3: Is the risk labelled or unlabeled?** | | | | | | | | | |
| *Unlabeled*  *(N = 750)* | *Labeled*  *(N = 750)* | *Unlabeled*  *(N = 750)* | | *Labeled*  *(N = 750)* | | *Unlabeled*  *(N = 750)* | | *Labeled*  *(N = 750)* | |
|  |  | **Randomization 4: Is the comparison presented in a relative or absolute way?**  *(Only for the COVID-19 and Motor vehicle mortality comparison)* | | | | | | | |
|  |  | *Abs*  *(N = 375)* | *Rel*  *(N = 375)* | *Abs*  *(N = 375)* | *Rel*  *(N = 375)* | *Abs*  *(N = 375)* | *Rel*  *(N = 375)* | *Abs*  *(N = 375)* | *Rel*  *(N = 375)* |
| Arm 2 | Arm 3 | Arm 4 | Arm 5 | Arm 6 | Arm 7 | Arm 8 | Arm 9 | Arm 10 | *Arm 11* |

The experimental text for each arm is:

**Arm 1:** With regards to side effects, so far 8 individuals have developed potentially life-threatening blood clots. This is among the approximately 7 million adults that have received the vaccine so far.

**Arm 2:** With regards to side effects, 1 out of 100,000 vaccinated individuals may develop serious blood clots.

**Arm 3:** With regards to side effects, 1 out of 100,000 vaccinated individuals may develop serious blood clots (very low risk).

**Arm 4:**

- *Text for USA participants:* With regards to side effects, 1 out of 100,000 vaccinated individuals may develop serious blood clots. As a reference, 170 out of every 100,000 unvaccinated Americans died of COVID-19 based on data from the past year.
- *Text for UK participants:* With regards to side effects, 1 out of 100,000 vaccinated individuals may develop serious blood clots. As a reference, 108 out of every 100,000 unvaccinated individuals in the UK died of COVID-19 based on data from the past year.

**Arm 5:**

- *Text for USA participants:* With regards to side effects, 1 out of 100,000 vaccinated individuals may develop serious blood clots. As a reference, this is 1/170th of the risk of COVID-19 mortality among unvaccinated Americans based on data from the past year.
- *Text for UK participants:* With regards to side effects, 1 out of 100,000 vaccinated individuals may develop serious blood clots. As a reference, this is 1/108th of the risk of COVID-19 mortality among unvaccinated individuals in the UK based on data from the past year.

**Arm 6:**

- *Text for USA participants:* With regards to side effects, 1 out of 100,000 vaccinated individuals may develop serious blood clots (very low risk). As a reference, 170 out of every 100,000 unvaccinated Americans died of COVID-19 based on data from the past year.
- *Text for UK participants:* With regards to side effects, 1 out of 100,000 vaccinated individuals may develop serious blood clots (very low risk). As a reference, 108 out of every 100,000 unvaccinated individuals in the UK died of COVID-19 based on data from the past year.

**Arm 7:**

- *Text for USA participants:* With regards to side effects, 1 out of 100,000 vaccinated individuals may develop serious blood clots (very low risk). As a reference, this is 1/170th of the risk of COVID-19 mortality among unvaccinated Americans based on data from the past year.
- *Text for UK participants:* With regards to side effects, 1 out of 100,000 vaccinated individuals may develop serious blood clots (very low risk). As a reference, this is 1/108th of the risk of COVID-19 mortality among unvaccinated individuals in the UK based on data from the past year.

**Arm 8:**

- *Text for USA participants:* With regards to side effects, 1 out of 100,000 vaccinated individuals may develop serious blood clots. As a reference, 12 out of every 100,000 Americans died in a motor vehicle accident based on data from the past year.
- *Text for UK participants:* With regards to side effects, 1 out of 100,000 vaccinated individuals may develop serious blood clots. As a reference, 2.6 out of every 100,000 individuals in the UK died in a motor vehicle accident based on data from the past year.

**Arm 9:**

- *Text for USA participants:* With regards to side effects, 1 out of 100,000 vaccinated individuals may develop serious blood clots. As a reference, this is 1/12th of the risk of dying in a motor vehicle accident based on data from the past year.
- *Text for UK participants:* With regards to side effects, 1 out of 100,000 vaccinated individuals may develop serious blood clots. As a reference, this is almost 1/4th of the risk of dying in a motor vehicle accident based on data from the past year.

**Arm 10:**

- *Text for USA participants:* With regards to side effects, 1 out of 100,000 vaccinated individuals may develop serious blood clots (very low risk). As a reference, 12 out of every 100,000 Americans died in a motor vehicle accident based on data from the past year.
- *Text for UK participants:* With regards to side effects, 1 out of 100,000 vaccinated individuals may develop serious blood clots (very low risk). As a reference, 2.6 out of every 100,000 individuals in the UK died in a motor vehicle accident based on data from the past year.

**Arm 11:**

- *Text for USA participants:* With regards to side effects, 1 out of 100,000 vaccinated individuals may develop serious blood clots (very low risk). As a reference, this is 1/12th of the risk of dying in a motor vehicle accident based on data from the past year.
- *Text for UK participants:* With regards to side effects, 1 out of 100,000 vaccinated individuals may develop serious blood clots (very low risk). As a reference, this is nearly 1/4th of the risk of dying in a motor vehicle accident based on data from the past year.

The risk information will be presented on a single screen along with the two main outcome questions. Lastly, for individuals that reported that they are unlikely or unsure about whether they would take the vaccine, the final page will ask them their reason (question based on a recently completed study of COVID-19 vaccine hesitancy).^12^

**Criteria for discontinuing or modifying allocated interventions {11b}**

Participants will only be discontinued if they voluntarily stop or withdraw their participation from the study.

**Strategies to improve adherence to interventions {11c}**

Not applicable.

**Relevant concomitant care permitted or prohibited during the trial {11d}**

Not applicable.

**Provisions for post-trial care {30}**

Not applicable.

**Outcomes {12}**

Our primary outcome is individuals' willingness to take the hypothetical COVID-19 vaccine. We will measure this outcome by asking, "How likely would you be to take this vaccine?" allowing individuals to choose from a four-point Likert response of "Unlikely, Unsure leaning towards unlikely, Unsure leaning towards likely, Very likely." This outcome variable, including the categories and phrasing, is based on a recently published study on COVID-19 vaccine hesitancy conducted by the Vaccine Hesitance Project at the London Schoon of Hygiene and Tropical Medicine.^13^ In our main analyses we will consider a dichotomous version of this outcome for whether individuals reported "Very likely" or not and assess the effect of the different framings on the proportion of respondents that reported "Very likely." As a sensitivity analysis, we will assess the effect of the framings on the un-dichotomized four category variable using an ordinal logistic regression model. The main effect of from this model will have the interpretation of the odds ratio of reporting more likely responses compared to less likely responses.

Our secondary outcome is individuals' perceived safety of the vaccine. We will assess this outcome by asking individuals, "How safe do you feel this vaccine is?" allowing them to choose answers ranging from 1-10 where 0 is extremely unsafe, and 10 is extremely safe. In our analyses, we will assess the effect of the different framings on mean reported safety.

**Participant timeline {13}**

We aim to begin recruitment on 26 July 2021 and end by August 10, 2021. We estimate the following overall study timeline:

| Item | May | June | July | August | September | October |
| --- | --- | --- | --- | --- | --- | --- |
| Obtain ethical clearance in Heidelberg |  |  |  |  |  |  |
| Register trial with pre-analysis plan on German Clinical Trials Register |  |  |  |  |  |  |
| Prepare and submit trial protocol for publication |  |  |  |  |  |  |
| Collect study data |  |  |  |  |  |  |
| Conduct data analysis and prepare manuscript for publication |  |  |  |  |  |  |

**Sample size {14}**

Our total sample is 12000 individuals, split evenly between the United States and the United Kingdom. We chose this sample size to enable us to detect a 5-percentage point difference between the experimental arms across the factor with the most groups (the comparison factor, which has three groups: (1) no comparison, (2) comparison to COVID-19 mortality, and (3) comparison to motor vehicle mortality). With 80% power, a 5% alpha value, and assuming a control group proportion of 50% (the most conservative assumption), this requires approximately 1500 individuals per arm. We additional added another 1500 respondents for the "status quo" framing, for a total sample of 6000 individuals per country.

**Recruitment {15}**

Participants will be recruited through Prolific.

**Assignment of interventions: allocation**

**Sequence generation {16a}**

Using a web-based randomization algorithm, Gorilla will randomly allocate participants to each of the experimental arms. Gorilla allows for two randomization options - independent randomization of each individual based on a probability draw and balanced randomization, which randomizes without replacement such that among groups of respondents a fixed proportion will end up in each experimental arm. We will use the "balanced randomization" option to ensure that our experimental arms are balanced.

**Concealment mechanism {16b}**

As the randomization is done by the Gorilla platform, neither the investigators nor the participants will have any access to the randomization mechanism.

**Implementation {16c}**

Implementation of the randomization will be done by the Gorilla platform.

**Assignment of interventions: Blinding**

**Who will be blinded {17a}**

Because Prolific handles the interaction between the study investigators and participants, the participants will be completely anonymous to the study investigators. The outcome measures will be self-reported and submitted anonymously. All persons in the study team will be blinded to the group allocation.

**Procedure for unblinding if needed {17b}**

Not applicable.

**Data collection and management**

**Plans for assessment and collection of outcomes {18a}**

Data will be collected and stored on the Gorilla platform. To ensure data quality, the study investigators conducted several trial runs of the experiment prior to finalizing design. Our primarily outcome variable is based on a recently completed study for measuring COVID-19 vaccine intentions.^13^

**Plans to promote participant retention and complete follow-up {18b}**

For the expected study duration of under 5 minutes, participants will be compensated £0.63/$0.87 for completing the experiment (those who do not complete the full experiment will not be compensated).

**Data management {19}**

Data will be collected and stored on the Gorilla platform. At the conclusion of the study, data will be downloaded to a secured folder only accessible by the study investigators.

**Confidentiality {27}**

All trial participants will be assigned a unique, anonymized string ID. The ID will be used on the Gorilla platform and linked to the participant’s responses. Gorilla will store the trial data on its cloud platform, hosted on Microsoft Azure in the Republic of Ireland. The Gorilla database is encrypted using industry-standard cryptography. The study investigators own the research data that has been collected using Gorilla and have complete control over it. The study investigators can generate and access the completely anonymized data from the Gorilla platform. The data will be downloaded and safely stored for statistical analysis on a computing system maintained by Heidelberg University in Germany.

We state that the names and all other personal and medical information are subject to confidentiality and to the regulations of the Federal Data Protection Act (Bundesdatenschutzgesetz – BDSG), the General Data Protection Regulation (Datenschutz-Grundverordnung – DSGVO) and State Data Protection Act (Landesdatenschutzgesetzes - LDSG). However, we stress again that we will not collect any personal information (such as the participant’s name) or medical information. Participants will be anonymous to the study investigators unless they email the study investigators. In this event, the study investigators will keep this information confidential.

**Plans for collection, laboratory evaluation and storage of biological specimens for genetic or molecular analysis in this trial/future use {33}**

Not applicable.

**Statistical methods**

**Statistical methods for primary and secondary outcomes {20a}**

We will estimate the impact of the different framings on our primary outcome using the following logistic regression models.

To estimate the effect of presenting the side-effect risk with a comparison and the effect of a qualitative risk label:

$$ln\left( \frac{\theta}{1-\theta} \right)=\alpha_{0}+\left( \alpha_{1}*CM_{i} \right)+\left( \alpha_{2}*MVM_{i} \right)+\left( \alpha_{3}*RL_{i} \right)+\boldsymbol{\zeta}$$

where $\theta$ is the probability of reporting "Very likely" to take the hypothetical vaccine, $CM_{i}$ is a binary indicator for whether individual *i* was randomized to receive a comparison with COVID-19 mortality, $MVM_{i}$ is a binary indicator for whether individual *i* was randomized to receive a comparison with motor vehicle mortality, $RL_{i}$ is an indicator for whether individual *i* was randomized to receive a qualitative risk label, and $\boldsymbol{\zeta}$ is a vector of covariates, including age, sex, education, and country.

To estimate the effect of absolute compared to relative comparisons, we will restrict our sample to only those who were randomized to receive a comparison and estimate the following regression:

$$ln\left( \frac{\theta}{1-\theta} \right)=\beta_{0}+\left( \beta_{1}*Rel_{i} \right)+\boldsymbol{\zeta}$$

where $Rel_{i}$ is a binary indicator for whether individual *i* was randomized to receive a relative comparison.

Lastly, as a secondary analysis, we will compare the effect of any informed risk-communication frame to the status-quo by estimating the following regression:

$$ln\left( \frac{\theta}{1-\theta} \right)=\delta_{0}+\left( \delta_{1}*Int_{i} \right)+\boldsymbol{\zeta}$$

where $Int_{i}$ is a binary indicator for whether individual *i* was randomized to any of the intervention arms.

For our secondary outcome (perceived safety of the vaccine), we will estimate these seem regressions using a linear, rather than logistic, model. For all logistic regressions, we will present coefficients as average marginal effects, rather than odds ratios, to ease interpretation.

**Interim analyses {21b}**

We will not conduct any interim analyses.

**Methods for additional analyses (e.g. subgroup analyses) {20b}**

We will determine if the size of the effects differs between the United States and United Kingdom by estimating additional models that interact the main effects with an indicator variable for country. We will additionally determine whether the effects vary by sex and broad age group using models that interact the main effects with indicator variables for female sex and whether an individual is above age 60.

**Methods in analysis to handle protocol non-adherence and any statistical methods to handle missing data {20c}**

As our intervention involves communication frames, non-adherence is not an issue. We will drop all individuals who do not provide responses to the main outcome measures.

**Plans to give access to the full protocol, participant level-data and statistical code {31c}**

We will post the de-identified data and statistical code in the Heidelberg University Open Data Repository after the publication of the main trial manuscript.

**Oversight and monitoring**

**Composition of the coordinating centre and trial steering committee {5d}**

The trial will be run online through the Gorilla platform and thus does not require a coordinating centre or trial steering committee.

**Composition of the data monitoring committee, its role and reporting structure {21a}**

The trial data will be automatically collected through the Gorilla platform and thus does not require a data monitoring committee.

**Adverse event reporting and harms {22}**

Our study does not involve the collection of any biomarker specimens, provision of interventions or treatments, nor clinical recommendations to participants. We also do not ask highly sensitive questions. For these reasons, we believe our study poses minimal risk to participants, and thus do not have an adverse event reporting system.

**Frequency and plans for auditing trial conduct {23}**

Not applicable.

**Plans for communicating important protocol amendments to relevant parties (e.g. trial participants, ethical committees) {25}**

We will communicate protocol changes to the German Clinical Trials Register and the Heidelberg University Ethics Commission. We will not make changes to protocol after the recruitment begins.

**Dissemination plans {31a}**

We will disseminate our results through presentations in academic conferences and through publication in a general medical peer reviewed journal.

**Discussion**

The results from this study will provide some of the first experimental evidence on how the communication of COVID-19 vaccine risks impacts willingness to be vaccinated and can inform ongoing vaccination efforts.

**Trial status**

Version 1.0, 14 July 2021. Recruitment has not yet begun and is anticipated to start on 26 July 2021.

**Abbreviations**

None.

**Declarations**

**Acknowledgements**

Not applicable.

**Authors’ contributions {31b}**

NS and AV conceived of the study. NS is the Chief Investigator and led the proposal and protocol development. All authors contributed to the study design and the development of the proposal and protocol. All authors read and approved the final manuscript.

**Funding {4}**

The study is funded by the Heidelberg Institute of Global Health, Heidelberg University. The funding body had no role in the design of the study and will have no role in the collection, analysis, and interpretation of the data nor in writing the manuscript.

**Availability of data and materials {29}**

Data will be collected and stored on the Gorilla platform. The study investigators own and have complete control of the research data, which can be accessed at any time. For statistical analysis, the data will be downloaded and safely stored on a computing system maintained by the University of Heidelberg.

**Ethics approval and consent to participate {24}**

The study received ethics approval from the Heidelberg University’s Ethics Committee on June 10th, 2021 (S-443/2021).

**Consent for publication {32}**

Not applicable.

**Competing interests {28}**

The authors declare that they have no competing interests.

**Authors’ information (optional)**

Not applicable*.*

**References**

1. New York Times. See How Vaccinations Are Going in Your County and State. New York Times. 2021. <https://www.nytimes.com/interactive/2020/us/covid-19-vaccine-doses.html?name=styln-vaccines-combo&region=TOP_BANNER&block=storyline_menu_recirc&action=click&pgtype=Interactive&variant=1_Show&is_new=false>. Accessed 13 Jul 2021.

2. Ritchie H, Ortiz-Ospina E, Beltekian D, Mathieu E, Hasell J, Macdonald B, Giattino C, Appel C, Rodés-Guirao L, Roser M. Coronavirus (COVID-19) Vaccinations. Our World in Data. 2021. <https://ourworldindata.org/covid-vaccinations>. Accessed 13 Jul 2021.

3. Centers for Disease Control and Prevention. CDC Recommends Use of Johnson & Johnson’s Janssen COVID-19 Vaccine Resume. 2021. <https://www.cdc.gov/coronavirus/2019-ncov/vaccines/safety/JJUpdate.html>. Accessed 6 Jun 2021.

4. European Medicines Agency (EMA). AstraZeneca’s COVID-19 vaccine: benefits and risks in context Share. European Medicines Agency. 2021. <https://www.ema.europa.eu/en/news/astrazenecas-covid-19-vaccine-benefits-risks-context>. Accessed 6 Jun 2021.

5. Bonner C, Trevena LJ, Gaissmaier W, Han PKJ, Okan Y, Ozanne E, Peters E, Timmermans D, Zikmund-Fisher BJ. Current Best Practice for Presenting Probabilities in Patient Decision Aids: Fundamental Principles. Med Decis Making. 2021. doi:10.1177/0272989X21996328.

6. Trevena LJ, Zikmund-Fisher BJ, Edwards A, Gaissmaier W, Galesic M, Han PK, King J, Lawson ML, Linder SK, Lipkus I, Ozanne E, Peters E, Timmermans D, Woloshin S. Presenting quantitative information about decision outcomes: a risk communication primer for patient decision aid developers. BMC Med Inform Decis Mak. 2013;13(S2):S7. doi:10.1186/1472-6947-13-S2-S7.

7. EUPATI. Risk communication in medicines. EUPATI. n.d. <https://toolbox.eupati.eu/resources/risk-communication-in-medicines/>. Accessed 6 Jun 2021.

8. Sinayev A, Peters E, Tusler M, Fraenkel L. Presenting Numeric Information with Percentages and Descriptive Risk Labels: A Randomized Trial. Med Decis Making. 2015;35(8):937–947. doi:10.1177/0272989X15584922.

9. Palm R, Bolsen T, Kingsland JT. The Effect of Frames on COVID-19 Vaccine Hesitancy. Health Policy. 2021. doi:10.1101/2021.01.04.21249241.

10. Prolific. 2021. <https://www.prolific.co/>. Accessed 14 Apr 2021.

11. Gorilla. 2021. <https://app.gorilla.sc/>. Accessed 14 Apr 2021.

12. Ruiz JB, Bell RA. Predictors of intention to vaccinate against COVID-19: Results of a nationwide survey. Vaccine. 2021;39(7):1080–1086. doi:10.1016/j.vaccine.2021.01.010.

13. Loomba S, de Figueiredo A, Piatek SJ, de Graaf K, Larson HJ. Measuring the impact of COVID-19 vaccine misinformation on vaccination intent in the UK and USA. Nat Hum Behav. 202;5(3):337–348. doi:10.1038/s41562-021-01056-1.
